# Severe Leptospirosis With Multi-Organ Dysfunction Syndrome in a Non-Endemic Region: A Case Report

**DOI:** 10.7759/cureus.104427

**Published:** 2026-02-28

**Authors:** Nikita S Jasani, Rana A Almugassabi, Rahaf A Alami, Tamer Mubarak, Asif M Salim

**Affiliations:** 1 Internal Medicine, GMERS Medical College and Hospital, Sola, Ahmedabad, IND; 2 Internal Medicine, Libyan International Medical University, Benghazi, LBY; 3 Internal Medicine, Yakın Doğu Üniversitesi, Lefkoşa, CYP; 4 Internal Medicine, Rashid Hospital, Dubai, ARE; 5 Internal Medicine, Dubai Hospital, Dubai, ARE

**Keywords:** encephalopathy, icteric leptospirosis, jaundice, leptospirosis, liver failure, multi-organ failure, rice farmers, septic shock, thrombocytopenia, weil’s disease

## Abstract

Leptospirosis is a common zoonotic infection worldwide but remains rare in the Middle East, including the United Arab Emirates. We report a case of a 36-year-old male who presented with high-grade fever, severe myalgia, headache, vomiting, dark urine, and altered mental status following recent travel from an endemic area where he worked as a paddy field farmer. Clinical examination and laboratory findings revealed septic shock, acute kidney injury, marked hyperbilirubinemia, and severe thrombocytopenia, consistent with multi-organ dysfunction syndrome. Based on the clinical presentation and exposure history, leptospirosis was strongly suspected, and *Leptospira *IgM serology was sent. The patient was managed in the intensive care unit with supportive care and broad-spectrum antibiotics, leading to gradual clinical improvement. This case highlights the need to consider leptospirosis in acute febrile illness with jaundice and organ dysfunction, even in non-endemic regions.

## Introduction

*Leptospira* are spiral-shaped, highly motile, aerobic, gram-negative bacteria that can be visualized using dark-field microscopy, silver staining, or fluorescent microscopy [1]. Though the disease is prevalent worldwide, it is concentrated in tropical and temperate climates. More than one million cases of leptospirosis are estimated worldwide annually, with almost 60,000 annual deaths attributed to this disease alone [2].

Mammals serve as the primary reservoir for *Leptospira*, and the environment can function as a secondary reservoir when contaminated by urine from infected mammals [1,2]. Rodents are the most common primary reservoirs, with others including cattle, swine, dogs, horses, sheep, and goats [1,3]. Once infected, *Leptospira *colonizes the renal tubules of these mammals, which subsequently shed the organism in their urine throughout their lifespan.

Humans are considered accidental hosts [4]. They are typically infected through contact with water, soil, or food contaminated by the urine of infected mammals or through direct contact with such urine, which is the most common route of transmission [1,5,6]. Transmission has also been reported via animal bites and, less commonly, through human-to-human routes such as sexual intercourse or breastfeeding [6]. Therefore, rice farmers, sewer workers, abattoir workers, veterinarians, and ranchers are at increased risk of contracting leptospirosis [1,2,7].

Transmission to humans occurs mainly through cuts or abraded skin, mucous membranes, or conjunctiva [1,6,7]. Clinical manifestations range from mild to severe [7]. The illness progresses through two main stages. The initial anicteric phase is typically mild, self-limiting, and lasts approximately 5-4 days [2], during which *Leptospira *circulate in the bloodstream (leptospirosis). In this phase, patients may experience constitutional symptoms such as fever, chills, headache, cough, muscle pain, anorexia, rash, and diarrhea. Some patients may progress to a secondary immune phase, presenting with aseptic meningitis, uveitis, cholecystitis, or pancreatitis [1,6-8].

The second stage, known as the icteric phase or Weil’s disease, represents the more severe form and may be life-threatening without prompt treatment [8,9]. This phase occurs in 5%-10% of cases and can rapidly progress to multisystem dysfunction, with mortality rates ranging from 5% to 15% [9]. Higher loads of leptospiremia activate innate immunity and a cytokine storm characterized by high levels of IL-6, IL-10, and TNF-alpha, which can trigger sepsis and multi-organ dysfunction [10]. Clinical features include fever, jaundice, renal failure, pulmonary hemorrhage with acute respiratory distress syndrome (ARDS), acute kidney injury, and rhabdomyolysis, hemorrhagic tendencies and hemolysis, respiratory compromise, sepsis, and, in advanced stages, multi-organ failure [1,6,7].

The clinical diagnosis is difficult due to a clinical presentation that mimics other infectious diseases, which may complicate early recognition. However, laboratory diagnosis is more straightforward, with cultures, serological assays, and molecular tests such as PCR being employed. Treatment depends on disease severity and may include doxycycline, penicillin, or third-generation cephalosporins [7,11].

In non-endemic regions, failure to recognize leptospirosis can lead to diagnostic delays and poor outcomes. This case reinforces the importance of incorporating travel and occupational history into the evaluation of febrile patients presenting with hepatic and renal dysfunction, progressing to sepsis and multi-organ dysfunction syndrome.

## Case presentation

Initial presentation and patient history

A 36-year-old previously healthy male, who recently arrived from an endemic region where he worked as a rice field farmer, presented to the emergency department of our hospital with a three-day history of high-grade fever, severe myalgia, headache, repeated vomiting, dark urine, and intermittent confusion.

Physical examination and hospital course

On arrival, he was febrile (38.4℃), hypotensive (83/48 mmHg), tachycardic (pulse rate of 126 beats/min), ill-appearing, visibly jaundiced, and clinically dehydrated. However, his respiratory rate was within an acceptable range, and he maintained an SpO_2_ of more than 96% on room air.

General examination revealed an ill-looking patient with dry mucous membranes, scleral and cutaneous jaundice, generalized myalgia, and headache. Abdominal examination demonstrated diffuse abdominal tenderness on palpation. The patient was admitted under the medical team for further workup and management.

The following day, the patient developed a petechial rash involving the trunk, upper limbs, and chest. Urine output was minimal, suggestive of acute renal failure. Respiratory systemic examination revealed bilateral posterior infra-scapular rales. Hemodynamic status improved on inotropic support, but the patient developed acute respiratory failure the same day, attributed to fluid overload due to aggressive fluid correction, and was transferred to the intensive care unit, where he was intubated and placed on mechanical ventilation and started on vasopressor support for septic shock.

On day four of hospitalization, the patient showed clinical improvement. Urine output returned to normal after initiation of continuous venous hemodiafiltration, and the patient became afebrile, with oxygen saturation maintained by mechanical ventilation. By day five, the patient was improving clinically, with pneumonia and septic shock resolving.

By day seven, the patient was weaned from mechanical ventilation and extubated, stepping down to a non-rebreather mask at 10 L/min. However, the patient’s condition deteriorated on day nine. He became tachypneic and tachycardic, appearing restless. The patient was deeply jaundiced, and neurological examination revealed confusion, delirium, mild agitation, and sluggish pupillary reactions bilaterally, attributed to hepatic encephalopathy. He also developed coagulopathy with raised PT, INR, and thrombocytopenia. At that time, the respiratory rate was 41 breaths/min, with an oxygen saturation of 97% on a nasal cannula.

On day 10 of hospitalization, the patient showed clinical improvement. He appeared comfortable, with no signs of acute distress. The patient was afebrile, with a regular pulse, stable blood pressure, and off vasopressor support. Respiratory examination revealed symmetrical air entry bilaterally, and the patient's vital signs were stable on supplemental oxygen, which was tapered to room air by day 12. The liver profile and mental status began to improve by day 13. The next day, renal dialysis was discontinued due to improved renal function, and the patient was transferred to the medical ward for supportive management.

**Figure 1:**
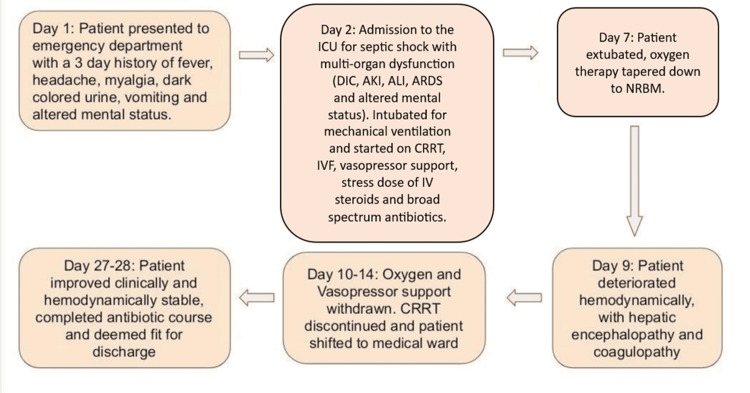
A summary of the patient's hospital course and clinical timeline ICU: Intensive care unit; AKI: Acute kidney injury; ALI: Acute liver injury; CRRT: Continuous renal replacement therapy; ARDS: Acute respiratory distress syndrome; NRBM: Non-rebreather mask.

One week before discharge, the patient had completed the prescribed course of antibiotics and continued to show clinical and biochemical improvement. He remained afebrile and hemodynamically stable, passing an adequate volume of urine. The respiratory exam was unremarkable with clear lung fields, and vital signs were stable. Renal and hepatic functions continued to improve with treatment, and septic markers remained within acceptable ranges. The patient was then deemed clinically fit for discharge. The patient’s clinical timeline since presentation is shown in Figure 1.

Laboratory investigations

Initial laboratory evaluation revealed significant abnormalities consistent with severe systemic inflammation evolving into multi-organ dysfunction. Hematological studies showed severe thrombocytopenia, marked neutrophilic leukocytosis, and normocytic normochromic anemia with reticulocytosis and raised lactate dehydrogenase (LDH), pointing to leptospirosis-related hemolysis in the absence of overt bleeding. Renal parameters demonstrated acute kidney injury with raised creatinine and decreased eGFR. Liver function tests revealed acute cholestatic liver injury with marked hyperbilirubinemia and elevated transaminases (Table 1).

**Table 1:** Laboratory investigations showing elevated septic markers, AKI, ALI, thrombocytopenia, and hemolytic anemia MCV: Mean corpuscular volume; MCH: Mean corpuscular hemoglobin; MCHC: Mean corpuscular hemoglobin concentration; RDW: Red cell distribution; AKI: Acute kidney injury; ALI: Acute liver injury; SGOT: Serum glutamic-oxaloacetic transaminase; SGPT: Serum glutamic-pyruvic transaminase.

Laboratory variables	Day 1 of admission	Day 9 of admission	Day of discharge	Laboratory variables
Complete blood count				
White blood cell count	22.8	29.1	8.5	3.6-11.0 x 10^3^/UL
Hemoglobin	10.9	7.2	9.1	13-17 g/dL
Red blood cell count	3.95	2.49	3.16	4.50-5.50 x 10^6^/UL
Hematocrit	32	20.6	27.6	%
MCV	81.2	82.8	87.3	77-95 FL
MCH	27.7	29.1	28.2	27-32 PG
MCHC	34.1	35.1	33	31.5-34.5 g/dL
RDW	13.2	16.8	16.9	11.5%-14.0%
Platelet count	9	80	332	150-410 x 10^3^/UL
Neutrophil absolute	20.98	14.12	3.5	2-7 x 10^3^/UL
Lymphocyte absolute	0.91	2.53	3.1	1-3 x 10^3^/UL
Monocyte absolute	0.46	0.36	1.2	0.20-1.00 x 10^3^/UL
Corrected reticulocyte count	5.41	-	-	0.5%-2.5%
Urea and electrolytes				
Urea	173	57	67	12-40 mg/dL
Potassium	4.5	4.5	4.3	3.4-5.0 mmol/L
Sodium	128	136	137	136-145 mmol/L
Chloride	87	102	101	98-108 mmol/
Ionized calcium	1.06	1.07	1.22	1.15-1.29 mmol/L
Ferritin	-	12,236	-	30-400 ng/mL
Ammonia	36	-	33	45 µmol/L
Glucose	118	96	91	60-100 mg/dL
Gamma-glutamyl transferase	52	238	-	8-61 U/l
Lactate	5.5	0.5	0.7	0.5-2.2 mmol/L
Renal function tests				
Creatinine	3.22	1.11	1.83	0.70-1.20 mg/dL
Estimated glomerular filtration rate	18.1	88.3	48.4	>60 mL/min/1.73 m
Inflammatory markers				
C-reactive protein	349	38.9	3.1	<5.0 mg/L
Procalcitonin	28.3	8.66	3.94	0.05 ng/mL
NT-pro B-type natriuretic peptide	31,507	25,404	2,798	<125 pg/ml
Liver function tests				
Total bilirubin	26.2	57.3	4.56	0-1.2 mg/dL
Alkaline phosphatase	109	190	163	40-129 U/L
Alanine aminotransferase (SGOT)	133	127	62	0-40 U/L
Aspartate aminotransferase (SGPT)	67	132	83	0-41 U/L
Albumin	3.4	3.7	3	4.4-5.1 g/dL
Globulin	3.6	2.6	3.3	2.8-3.4 g/dL
Coagulation profile				
Prothrombin time	10.3	14.3	10.6	9.7-11.8 s
International normalized ratio (INR)	0.98	1.37	1.01	0.8-1.1
Activated partial thromboplastin time (APTT)	43.9	58.2	36.7	25.1-37.7 s
Fibrinogen	684.2	632.77	539.59	200-400 mg/dL
Other laboratory investigations				
Lactate	306	675	-	105-222 mmol/L
Creatine phosphokinase (CPK)	3,827	-	17	<190 U/L
Blood culture	Negative	-	Negative	Negative

Serum lactate increased significantly within the first 24 hours, indicating worsening tissue hypoperfusion in the background of sepsis. Inflammatory markers were profoundly elevated, consistent with severe sepsis. Elevated NT-pro BNP was most likely related to sepsis-induced cytokine stimulation, renal impairment, vasopressor use, volume overload, and cardiac strain. Raised ferritin level corresponded to an acute inflammatory response, being an acute phase reactant, while elevated creatine phosphokinase (CPK) supported leptospiral-associated myositis (Table 1).

Clinical syndrome, occupational history, epidemiological exposure, positive serology, and exclusion of other differentials by serological testing and cultures (Table 2) strongly supported the diagnosis of leptospirosis, but it was not confirmed by molecular methods.

**Table 2:** Serological and immunological investigations PCR: Polymerase chain reaction; HIV: Human immunodeficiency virus; HTLV: Human T-lymphotropic virus; Ig: Immunoglobulin.

Serological and immunological investigations		
*Leptospira*IgG antibodies	<2.0	<10 U/mL
*Leptospira *IgM antibodies	54.3	<15 U/mL
Malaria parasite blood film	Negative	Negative
Dengue virus RNA PCR	Negative	Negative
Blood group	B+	-
Hepatitis A virus IgM antibody	Negative	Negative
Hepatitis B surface antigen	Negative	Negative
Hepatitis C antibodies	Negative	Negative
HIV 1 and 2 antigens and antibodies	Negative	Negative
Mycoplasma pneumonia IgG	Negative	Negative
Mycoplasma pneumonia IgM	Negative	Negative
Crimean-Congo hemorrhagic fever virus RNA PCR	Negative	Negative
Respiratory cultures	Negative	Negative
Pneumonia panel	Negative	Negative
Tuberculosis PCR	Negative	Negative
Acid-fast bacilli smear and culture	Negative	Negative
*Rickettsia rickettsii* and *Rickettsia typhi* antibodies, IgM and IgG	Negative	Negative
*Brucella *blood culture	Negative	Negative
*Coxiella burnetii *IgG and IgM antibodies	Negative	Negative
Cytomegalovirus DNA PCR	Negative	Negative
Epstein-Barr virus DNA PCR	Negative	Negative
HTLV 1 and 2 antibodies	Negative	Negative
Double-stranded DNA antibodies	<10	>100 UI/mL
Extractable nuclear antigen (ENA) scleroderma	Negative	Negative
Rheumatoid factor	Negative	<20 IU/mL
Antimitochondrial M2 antibody (AMA-M2)	Negative	<0.1 Negative
Antinuclear antibodies	Negative	Negative
Reticulocyte count	7.62%	0.5%-2.5%
Interleukin 2 receptor	Negative	Negative
Protein creatinine ratio	294	<50%

Urine analysis on admission showed hematuria, which improved gradually with continuous renal replacement therapy (Table 3). 

**Table 3:** Urine analysis on admission

Laboratory variables	Day of admission	Reference range
Urine color	Amber	Yellow
Urine clarity	Clear	Clear
Urine pH	7	5.0-7.5
Urine protein	2+	Negative
Urine glucose	2+	Negative
Ketones	Negative	Negative
Bilirubin	3+	Negative
Urobilinogen	1+	Normal
Nitrite	Negative	Negative
Leukocyte esterase	Negative	Negative
Specific gravity	1.011	1.002-1.030
White blood cells per high-power field	15-20	0-5
Red blood cells per high-power field	10-15	0-2

Imaging

Chest Radiograph

**Figure 2:**
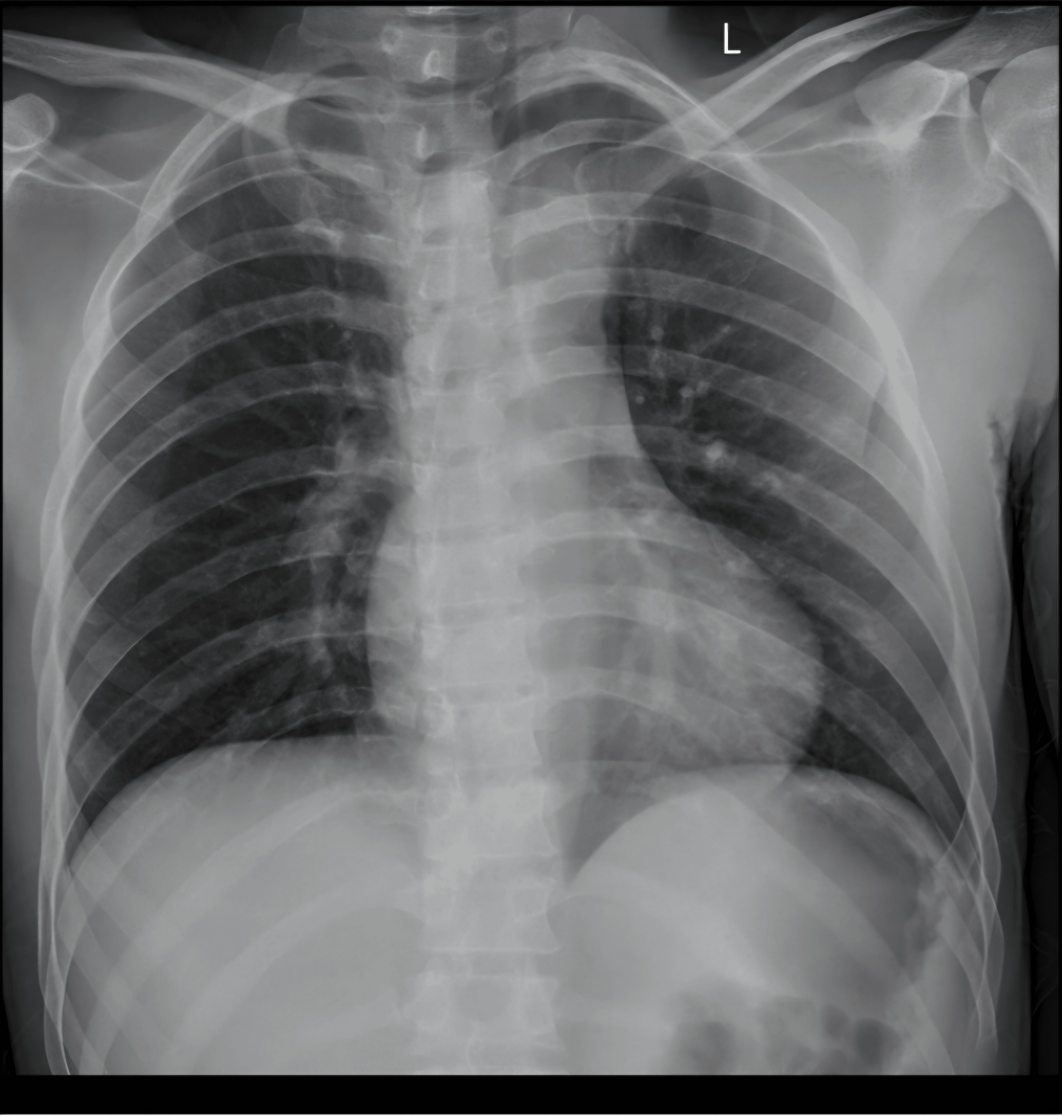
Chest radiograph with normal findings on the day of admission

Chest radiograph (Figure 2) obtained on admission demonstrated clear lung fields with no evidence of focal consolidation, collapse, pleural effusion, or pneumothorax. Cardiac size was within normal limits, and the mediastinal and hilar contours were unremarkable. No acute osseous abnormalities were identified.

**Figure 3:**
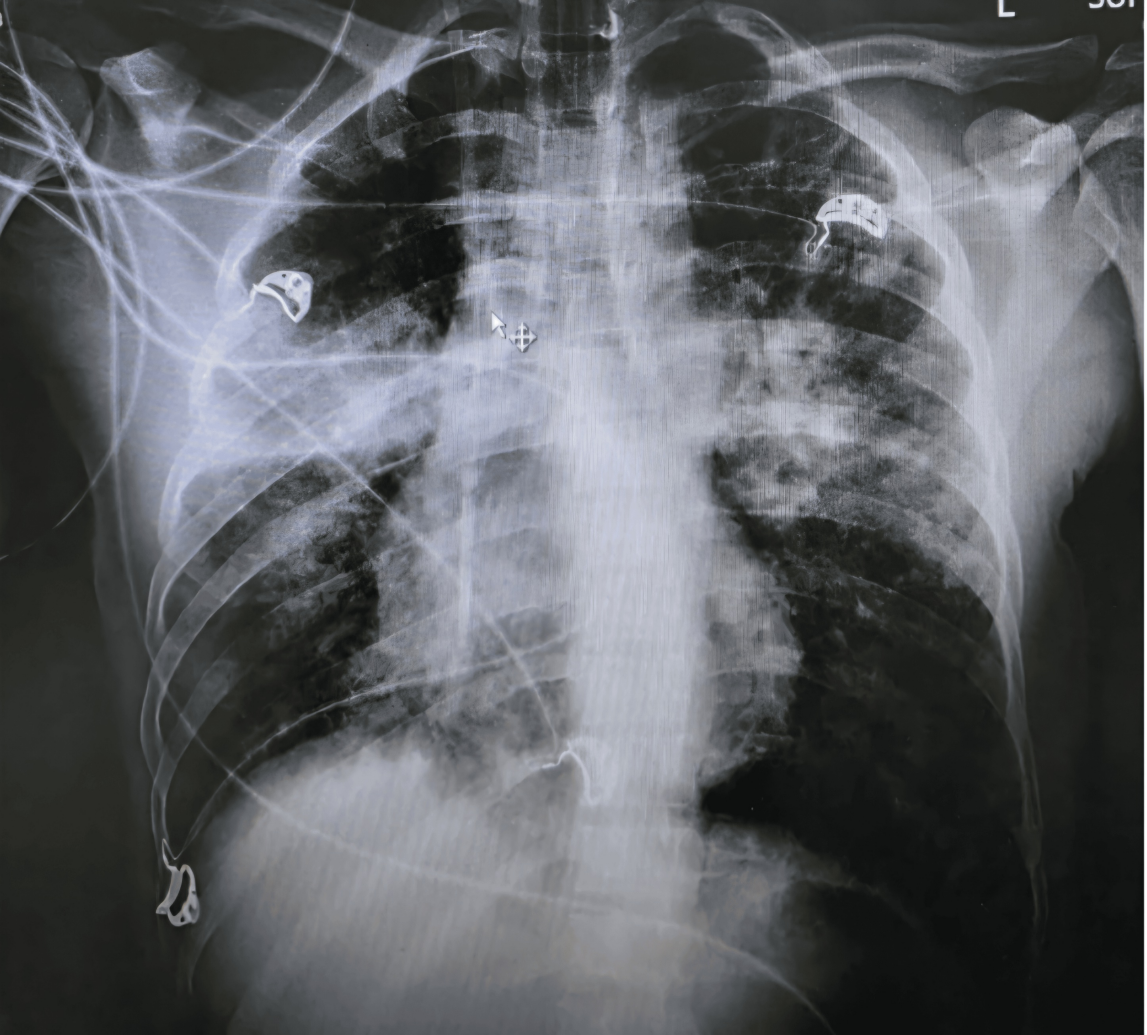
Chest radiograph showing fluid overload pulmonary edema and pleural effusion one day after admission

The chest X-ray performed one day later (Figure 3) was suggestive of pulmonary edema with patchy infiltrates involving both upper lobes and the right lower lobe, with fluid in the right horizontal fissure, suggesting a pleural effusion. The pulmonary edema was attributed to AKI and fluid overload on day two of admission. Pleural effusion was most likely due to sepsis-induced capillary leak, as a sequelae of severe leptospirosis induced cytokine storm and endothelial injury, which gradually improved with supportive management by day nine.

**Figure 4:**
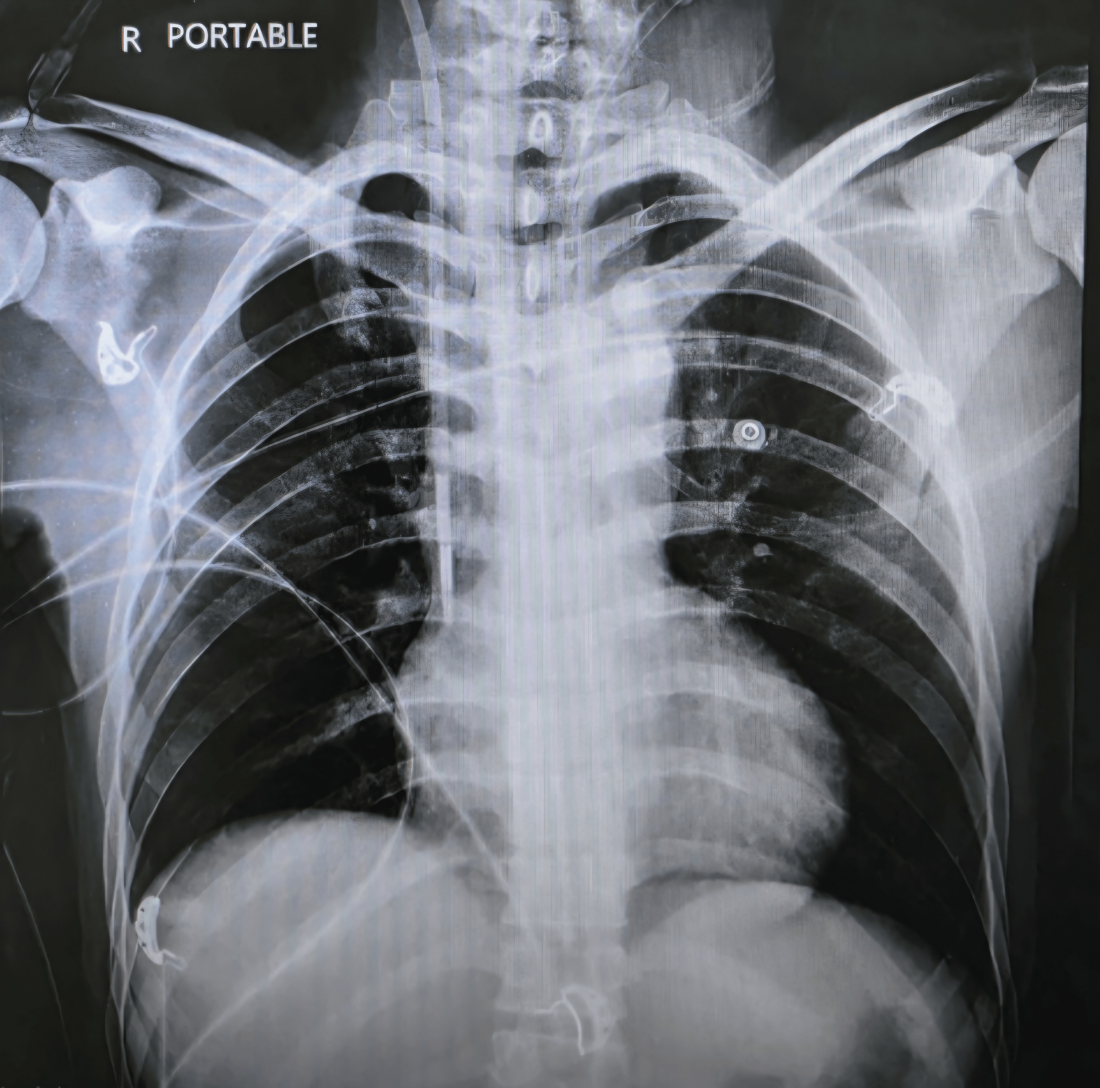
Follow-up chest radiograph on day nine showing resolution of pleural effusion and pulmonary edema

On day nine of admission, the chest X-ray (Figure 4) showed prominent bronchial vascular markings with resolved pleural effusion and pulmonary edema.

Transthoracic Echocardiography

Transthoracic echocardiography showed normal left ventricular size and systolic function with an ejection fraction of 55%-60% and no regional wall motion abnormalities. Diastolic function was normal. The right ventricular size and function were preserved. Cardiac valves were found to be structurally standard with only mild tricuspid and pulmonary regurgitation. There was no pericardial effusion. The inferior vena cava was collapsible with respiration. A dilated superior vena cava was noted, and a bubble study was recommended to exclude a persistent left-sided superior vena cava.

Abdominal Ultrasound

**Figure 5:**
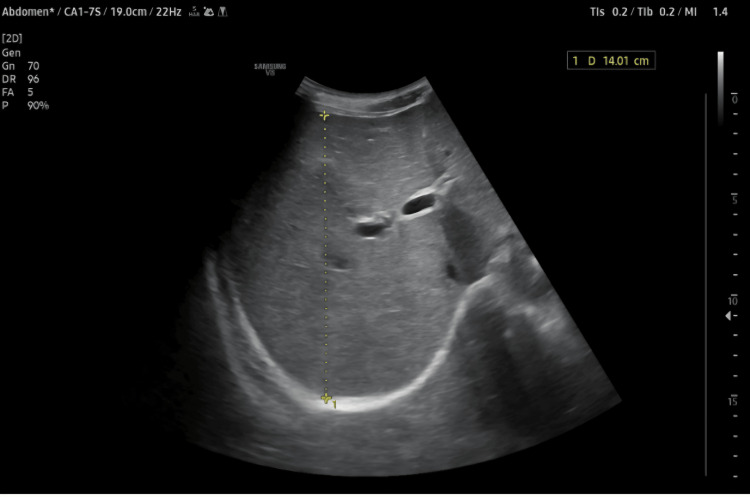
Mild hepatomegaly and prominent hepatic veins on abdominal ultrasound

Ultrasound of the abdomen on the day of admission revealed mild hepatomegaly with prominent hepatic veins, without focal hepatic lesions or intrahepatic or extrahepatic biliary ductal dilatation (Figure 5).

**Figure 6:**
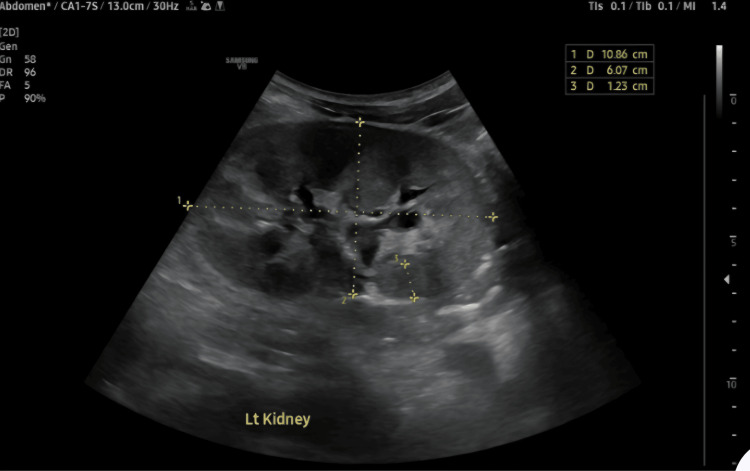
Ultrasonography of the left kidney showing increased cortical echogenicity and mild impairment of corticomedullary differentiation

Both kidneys demonstrated increased cortical echogenicity with mildly impaired corticomedullary differentiation, consistent with bilateral grade 3 renal parenchymal disease (Figure 6). No hydronephrosis, renal calculi, or focal renal lesions were identified. No ascites were noted.

**Figure 7:**
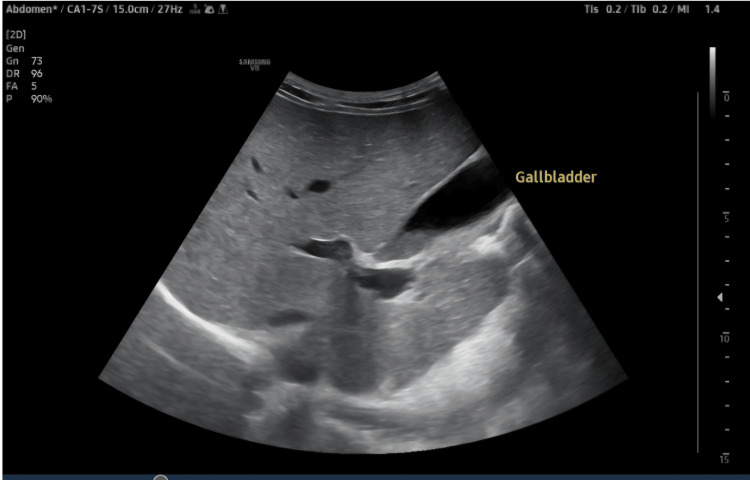
Gall bladder filled with sludge, showing mild wall thickening with pericholecystic edema

The gallbladder was filled with sludge and showed mild wall thickening with pericholecystic edema. The pancreas and spleen were unremarkable (Figure 7).

Follow-up abdominal ultrasound on day 17 of admission demonstrated persistent mild hepatomegaly with diffusely increased hepatic echogenicity, suggestive of fatty infiltration. There was an interval improvement in gallbladder findings, with a reduction in intraluminal sludge and complete resolution of the previously noted wall thickening and pericholecystic edema. Both kidneys remained mildly enlarged with persistent increased cortical echogenicity and impaired corticomedullary differentiation, consistent with ongoing bilateral renal parenchymal disease. No hydronephrosis or focal renal lesions were observed. No ascites were present.

Magnetic Resonance Cholangiopancreatography (MRCP)

**Figure 8:**
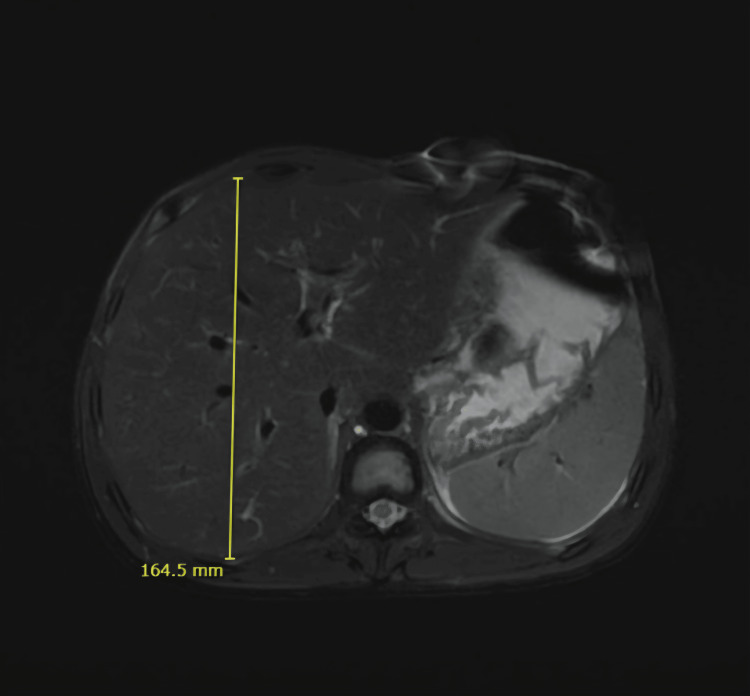
Mild hepatosplenomegaly on magnetic resonance cholangiopancreaticography

MRCP done on day 17 demonstrated mild hepatosplenomegaly without focal hepatic lesions or intrahepatic or extrahepatic biliary ductal dilatation. Patchy, ill-defined areas of mild hyperintensity were noted within the liver parenchyma on diffusion-weighted imaging, suggestive of inflammatory parenchymal changes (Figure 8).

**Figure 9:**
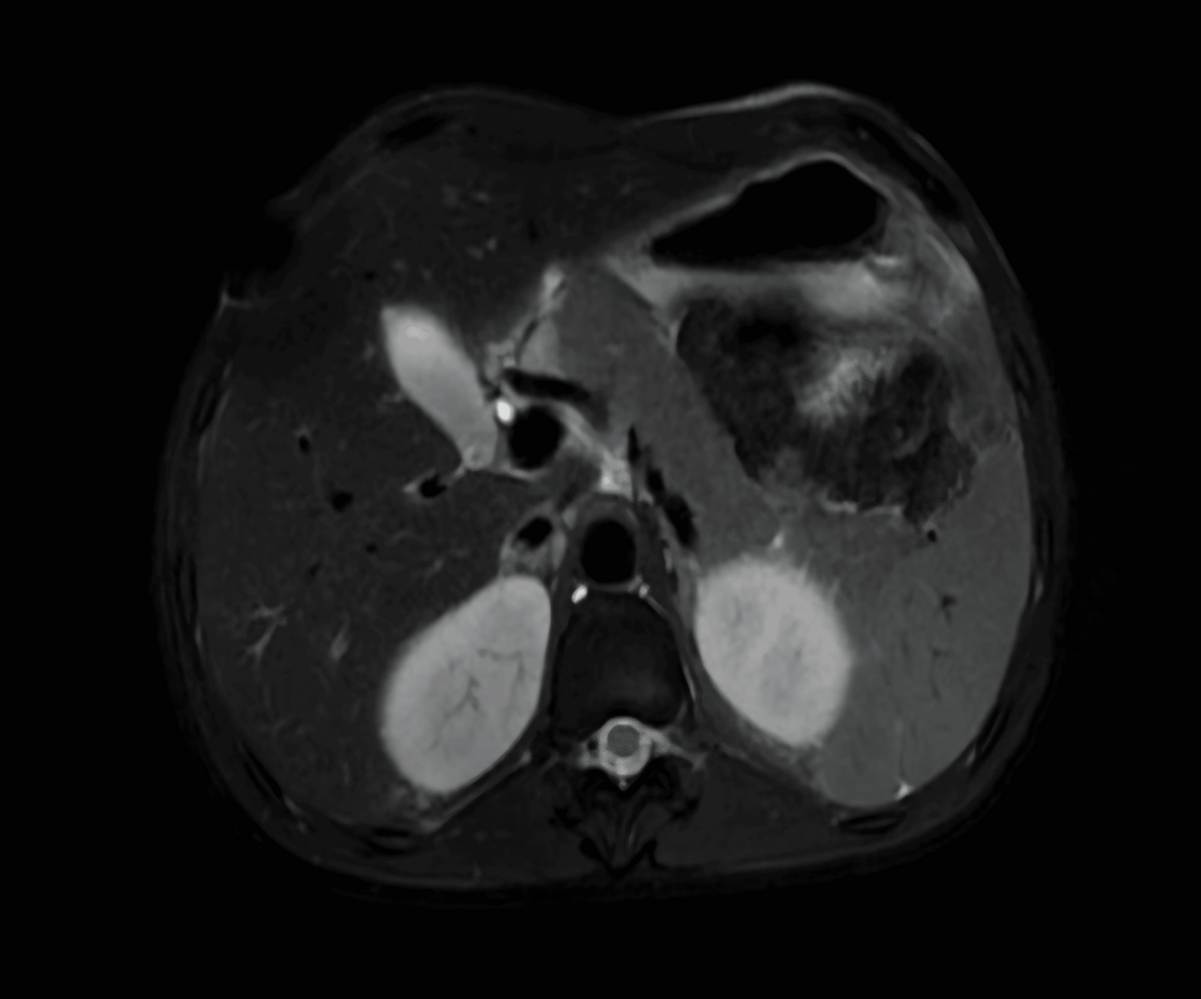
Gall bladder sludge with calculus in the gall bladder neck on magnetic resonance cholangiopancreaticography

The gallbladder contained sludge with a tiny calculus in the neck (Figure 9). Both kidneys appeared bulky with minimal perinephric fluid, but no evidence of hydronephrosis or focal lesions was found. Additional findings included mild bilateral pleural effusions with basal atelectasis (right greater than left), minimal free fluid in the perihepatic and peri-splenic regions, and subcutaneous soft-tissue edema.

Differential diagnosis

This particular patient had a positive travel history to an endemic zone and occupational exposure to paddy fields in Bangladesh. He had constitutional symptoms like fever, myalgia, rash, jaundice, and vomiting, with laboratory investigations revealing leukocytosis, thrombocytopenia, anemia, acute kidney injury, hyperbilirubinemia, and high septic and inflammatory markers.

Patient history, physical examination, and laboratory investigations raised suspicion of infectious causes, including leptospirosis, dengue, malaria, viral hepatitis, Crimean-Congo hemorrhagic fever, rickettsia, brucellosis, Q fever, typhoid fever, and human immunodeficiency virus (HIV) infection [10,12]. Autoimmune diseases were also ruled out by serological tests to be thorough, but leptospirosis emerged as the diagnosis based on clinical history, physical examination findings, and laboratory investigations.

Treatment

He was admitted to the ICU with a diagnosis of septic shock, acute kidney injury, and acute liver injury, where he received aggressive intravenous fluid resuscitation, vasopressor support, and platelet transfusions while on mechanical ventilation. A stress dose of intravenous hydrocortisone (150 mg TDS) was started on day two for vasopressor-dependent septic shock on adequate fluid resuscitation, to maintain a mean arterial pressure > 65 mmHg, and continued for six days till the patient clinically improved and was subsequently weaned off vasopressor support on day 10.

The infectious diseases unit decided to start doxycycline 100 mg BD (twice a day) to cover for atypical organisms and leptospirosis. Meropenem 1 g TDS (three times a day) was initiated as empirical broad-spectrum therapy due to severe sepsis with multi-organ dysfunction and an unclear infectious source at presentation. Once the diagnosis of leptospirosis was supported by positive IgM serology, therapy was appropriately de-escalated to doxycycline 100 mg BD and ceftriaxone 2 g OD (once a day), in accordance with severe leptospirosis treatment guidelines. He was weaned off mechanical ventilation and extubated on day seven of admission and stepped down to supplemental oxygen support. Once hemodynamically stable, clinically improving, and lab investigations trending toward the normal range, he was shifted to the medical ward on day 14.

Due to progressive renal deterioration and metabolic acidosis, he was started on continuous renal replacement therapy (CRRT) on day two of admission. He also required multiple platelet and packed red blood cell transfusions for severe thrombocytopenia and non-immune hemolytic anemia. Deoxycholic acid and supportive measures were continued for his hyperbilirubinemia.

Outcome and follow-up

His clinical course gradually improved. CRRT was discontinued three weeks later as his renal function began recovering. Platelet count, bilirubin, and transaminase levels were improving, and he regained adequate urine output. Throughout admission, all cultures remained negative.

By the end of his 28-day hospital course, the patient was afebrile, alert, hemodynamically stable, on room air with no supplemental oxygen requirement, tolerating oral intake, producing adequate urine output, and showing consistent biochemical improvement. Once clinically improving and vitally stable, he was considered fit for discharge on oral medication following transfusion of one unit of packed red cells, with instructions for outpatient follow-up with nephrology and gastroenterology for continued renal and hepatic recovery.

## Discussion

Leptospirosis remains a frequently overlooked illness, emphasizing the need for clinicians to maintain a high level of suspicion, especially in travelers from endemic regions. Timely recognition, the initiation of empirical antibiotic therapy, and proper supportive care are key factors that can improve patient outcomes, even in severe presentations [10,11]. The majority of reported cases originate from tropical and subtropical regions such as Southeast Asia, Latin America, and sub-Saharan Africa, with sporadic cases reported from the Middle East, where most documented cases are travel-related [7,9,12].

In our case, the simultaneous presentation of fever, jaundice, and acute kidney injury fulfilled the classical triad of severe leptospirosis (Weil's disease), while thrombocytopenia and hemodynamic instability further indicated systemic involvement and disease severity.

The patient in this report worked in a region that was heavily infested with rodents, with exposure occurring in early November, at the end of the monsoon season [6,7]. Few epidemiological studies found that agricultural exposure, particularly rice farming, is one of the strongest risk factors for leptospirosis [3,4]. Seasonal peaks following monsoon rainfall have been well described in endemic regions and are attributed to flooding and increased environmental contamination by rodent urine [5,6].

The pathogenesis of leptospirosis is well documented [1,2,4,6,8,13]. Once the *Leptospira *organism enters the host, it circulates through the bloodstream, producing bacteremia. Through hematogenous spread, it localizes in multiple organs, most commonly the liver, kidneys, spleen, and lungs, and establishes infection. IgM antibodies to *Leptospira *typically reach their peak around the fifth day after infection. The incubation period is generally 5-14 days, but it can range from 2 to 30 days in some cases [6,8,13].

The nonspecific clinical presentation of leptospirosis frequently leads to misdiagnosis as dengue, malaria, viral hepatitis, or other tropical infections [7,10]. In non-endemic regions, this diagnostic challenge is amplified. While molecular diagnostics such as PCR are most sensitive during early infection, serological testing remains the most widely available diagnostic modality [1,7]. In this case, early serological testing combined with strong clinical suspicion enabled confirmation without significant diagnostic delay.

Empirical antibiotic therapy is recommended as soon as leptospirosis is suspected [13]. For mild cases, doxycycline (100 mg orally twice daily for seven days) or azithromycin (500 mg once daily for three days) is recommended. In severe infections, intravenous options such as ampicillin (0.5-1 g every six hours), penicillin (1.5 million units four times daily), ceftriaxone (1 g daily), or cefotaxime (1 g four times daily for seven days) are indicated [10,11,14]. Other studies demonstrated that early initiation of appropriate antimicrobial therapy reduces disease severity and duration [10,11]. Ceftriaxone, doxycycline, and penicillin have comparable efficacy in severe disease [11,14].

Despite the presence of multiple poor prognostic indicators, including septic shock, renal failure requiring CRRT, and severe thrombocytopenia, the patient achieved complete clinical stabilization with gradual organ recovery. This outcome contrasts with historical mortality rates of 10%-15% reported for Weil’s disease and highlights the impact of early recognition, aggressive supportive care, and advanced critical care resources [7,8].

Several case series from non-endemic regions emphasize that low regional incidence often leads to delayed diagnosis and worse outcomes [7,12]. Prompt clinical suspicion and early initiation of antimicrobial and supportive management likely contributed to the favorable outcome in our case.

A limitation of this case is that confirmatory tests for leptospirosis, such as PCR or the microscopic agglutination test (MAT), were not performed. Therefore, diagnosis relied on clinical presentation, exclusion of other diagnoses, and positive IgM serology, which may be associated with false-positive results, particularly in the setting of severe systemic inflammation.

## Conclusions

This report describes a case of severe icteric leptospirosis (Weil’s disease) in a previously healthy young individual, presenting with septic shock, acute kidney injury requiring renal replacement therapy, profound hyperbilirubinemia, thrombocytopenia, encephalopathy, and respiratory involvement. Recent travel to an endemic area and occupational exposure were key epidemiological clues that guided early diagnostic consideration.

In non-endemic regions, leptospirosis remains underdiagnosed and may be mistaken for other tropical infections such as dengue or malaria, leading to delays in targeted therapy. This report contributes to the limited literature on severe leptospirosis in the Middle East and the need to consider leptospirosis in patients with acute febrile illness and multi-organ dysfunction regardless of geographic setting. Timely antimicrobial therapy and comprehensive supportive care can lead to favorable outcomes.

## References

[1] Day NPJ (2026). Leptospirosis: epidemiology, microbiology, clinical manifestations, and diagnosis. https://www.uptodate.com/contents/leptospirosis-epidemiology-microbiology-clinical-manifestations-and-diagnosis.

[2] (2026). LEPTOSPIROSIS fact sheet for clinicians. https://stacks.cdc.gov/view/cdc/52537/cdc_52537_DS1.pdf.

[3] Brockmann SO, Ulrich L, Piechotowski I, Wagner-Wiening C, Nöckler K, Mayer-Scholl A, Eichner M (2016). Risk factors for human Leptospira seropositivity in South Germany. Springerplus.

[4] Ko AI, Goarant C, Picardeau M (2009). Leptospira: the dawn of the molecular genetics era for an emerging zoonotic pathogen. Nat Rev Microbiol.

[5] Pothuri P, Ahuja K, Kumar V, Lal S, Tumarinson T, Mahmood K (2016). Leptospirosis presenting with rapidly progressing acute renal failure and conjugated hyperbilirubinemia: a case report. Am J Case Rep.

[6] Samrot AV, Sean TC, Bhavya KS (2021). Leptospiral infection, pathogenesis and its diagnosis-a review. Pathogens.

[7] Haake DA, Levett PN (2015). Leptospirosis in humans. Curr Top Microbiol Immunol.

[8] Plank R, Dean D (2000). Overview of the epidemiology, microbiology, and pathogenesis of Leptospira spp. in humans. Microbes Infect.

[9] Pavli A, Maltezou HC (2008). Travel-acquired leptospirosis. J Travel Med.

[10] Chacko CS, Shravya S, Jayakumar A (2021). A short review on leptospirosis: clinical manifestations, diagnosis and treatment. Clin Epidemiol Glob Health.

[11] McBride JA, Athanazio DA (2026). Leptospirosis: treatment and prevention. http://ttps://www.uptodate.com/contents/leptospirosis-treatment-and-prevention.

[12] Bandara M, Ananda M, Wickramage K, Berger E, Agampodi S (2014). Globalization of leptospirosis through travel and migration. Global Health.

[13] Eddicks M, Gründl J, Seifert A (2023). Examination on the occurrence of coinfections in diagnostic transmittals in cases of stillbirth, mummification, embryonic death, and infertility (SMEDI) syndrome in Germany. Microorganisms.

[14] Vinholo TF, Ribeiro GS, Silva NF, Cruz J, Reis MG, Ko AI, Costa F (2020). Severe leptospirosis after rat bite: a case report. PLoS Negl Trop Dis.

